# Social Workers’ Perspectives on Strengthening Parents in the Context of Inpatient Addiction Rehabilitation. A Reflexive Thematic Analysis

**DOI:** 10.2147/SAR.S536038

**Published:** 2025-09-16

**Authors:** Lena Mahnke, Ananda Stullich, Laura Hoffmann

**Affiliations:** 1 Department Health and Sport Sciences, Technical University of Munich, Munich, Germany

**Keywords:** social work, substance use disorder, stationary rehabilitation, family-orientation, parenting, intervention

## Abstract

**Purpose:**

In inpatient addiction rehabilitation, the context factors of parenthood and family have long been neglected, and evidence-based inpatient, standardized programs to strengthen rehabilitants in their role as parents are not routinely implemented in Germany. Social workers are highly involved in the field of addiction and youth welfare support. Therefore, this study aimed to capture their perspectives on how to strengthen parents in the context of an inpatient addiction rehabilitation intervention.

**Methods:**

Semi-structured expert interviews were conducted with social workers working with parents with substance use disorder (SUD) in ambulatory settings. The interviews were transcribed verbatim and analyzed using Reflexive Thematic Analysis.

**Results:**

The analysis identified four main themes that are crucial for strengthening rehabilitants in their parental role. Theme one deals with the exploration of different spheres of parenthood as intervention content: the subjective perception as a parent, the parental role as a child carer, and as family manager. Theme two concerns the methods through which (theoretical knowledge transfer, reflexive and practical exercise) and social settings in which (single, one-to-one, and group settings) this intervention content shall be provided. In theme three, the importance of a sensitive and cooperative professional attitude of rehabilitation associated professional health and social care workers – towards rehabilitants and professionals alike – is included. Theme four stresses the need for a family-centered rehabilitation organization by working as a professional interinstitutional network and arranging adequate childcare.

**Conclusion:**

Results show that social workers see substantial potential in inpatient addiction rehabilitation for strengthening parents with SUD in their parenting role. The multifaceted parental role should be examined in depth during rehabilitation in a trusting atmosphere. After discharge, especially (social pedagogical) follow-up care is considered crucial for perpetuating insights and progressions and sustainably strengthening rehabilitants, thus, the family system as a whole.

## Introduction

Worldwide, 2.6 million deaths were attributed to alcohol consumption (which accounts for 4.7% of all deaths), and 0.6 million deaths were linked to psychoactive drug usage in 2019, as the latest report of the World Health Organization (WHO) highlights.^1^ Substance Use Disorder (SUD) is one of the causes of these preventable deaths and creates a high individual and societal burden. While direct and indirect health and economic costs are calculable, intangible costs for communities and families remain massively neglected. In the social surroundings, especially noticeable and burdensome is the disease for people close to the person with SUD, like their partner, relatives, and friends, and especially children, who generally depend on their parents’ care.^2^,^3^ In the U. S. combined results of the 2009 to 2014 National Surveys on Drug Use and Health suggest that about 10.5% of the children aged 17 and younger (7.5 million) lived in households where at least one parent had an alcohol use disorder and approximately 2.9% of the children in this age group (2.1 million) lived in households with at least one parent with an illicit drug use disorder within the past year.^4^ Estimates in German studies came to similar results. Kraus et al concluded that 5.1–9.2% (688,111–1,257,345) of the children in 2018 lived in a household with at least one adult suffering from alcohol-related use disorder and 0.6–1.2% (87,817–158,401) in a household with at least one adult with illicit drug-related use disorder (according to DSM-IV diagnostic criteria).^5^ Due to different methodological approaches and the difficulties in collecting this kind of sensitive data, reliable data is scarce, and it can be assumed that the actual number of children affected is much higher than the existing estimates.^6^

Parents with SUD more frequently show inappropriate, harmful, and/or traumatizing parenting behavior than parents without SUD^7^ eg, by neglecting parental obligations and various forms of abuse.^6^ Unstable domestic circumstances can, also if unintended, and often unrecognized by parents^8^ endanger the healthy physical, psychological, and social development of underaged children.^7^,^8^ This can lead to lifelong negative consequences.^9^ Overall, the likelihood of developing psychological, behavioral, and socially relevant disturbances during the upbringing of these children is increased (eg, the likelihood of affective and personality disorders is 80% higher with a drug-dependent parent) compared to children of the same age having parents without SUD.^10^,^11^

The WHO urgently calls for more actions globally to achieve the Sustainable Development Goal target 3.5 of strengthening the prevention of substance abuse and improving high-quality SUD treatment until 2030.^1^ In an international comparison Germany provides a complex and multidisciplinary support structure for people with SUD within ambulatory, partially inpatient, as well as inpatient settings. Normally, after ambulatory counseling and inpatient detoxification, the treatment of patients with SUD continues in medical inpatient addiction rehabilitation (in short: rehabilitation). This rehabilitation lasts 3–6 months,^12^ in which rehabilitants should be stabilized and prepared for an abstinent everyday life, including employment. Currently, in inpatient addiction rehabilitation, evidenced-based interventions targeting explicitly strengthening parents in their parental role by including the contextual factors of family and parenthood within the rehabilitation process are nationwide not routinely implemented. Also in the US SUD-related collaborative care models for chronic disease management postpartum are insufficient. Most of the existing interventions focus solely on mothers,^13–18^ and most are designed for an outpatient or home setting.^19^ Considering parental duties’ significant impact – also as a reoccurring stressor^20^,^21^ – on everyday life, it is highly crucial to strengthen parents with SUD in their parental role.^22^,^23^ Inpatient rehabilitation settings provide a secure and safe living environment without pressures and distractions that other settings often include.^19^ This is why this study explores the inpatient setting more in depth for intervention conductance for mothers and fathers with SUD alike.

The intervention AddictionContext (in German KontextSucht Intervention (in the following: KSI)) aims to support parents with SUD in fostering their parental competencies during inpatient rehabilitation and is scientifically evaluated by the Chair of Social Determinants of Health at the Technical University of Munich. Detailed information can be found in the respective study protocol.^24^ The project is part of the funding line “rehapro” which is financially supported by the German Federal Ministry of Labor and Social Affairs.^25^

Social workers are highly involved in general and family-oriented ambulatory addiction^26^ support and in child and youth welfare services eg, in support for upbringing via parenting counseling and social pedagogical family assistance.^27^,^28^ Thereby, professionals aim for person-centered support for the parent with SUD and the safety and well-being of the child/children living together or being affiliated with that parent.^23^ Regardless of this high involvement of social workers in the ambulatory care of patients before and often after rehabilitation, until now, social workers’ perspectives concerning an effective intervention for strengthening parents in inpatient rehabilitation remain unexplored. Due to their expertise and insight into the living reality and needs of the affected parents as well as the children,^29^ social workers have promising perspectives and valuable insights for understanding potential success conditions of interventions aiming for strengthening rehabilitants in their parental role. The aim of this study is to capture explicitly these social workers’ perspectives on how to strengthen parents in the context of inpatient addiction rehabilitation. Informed by study results, interventions can be more adequately tailored to support rehabilitants in their parenting role, foster parenting competencies, and effectively prepare them for daily family life following inpatient rehabilitation.

## Materials and Methods

### Study Design

An explorative qualitative study design based in an experiential camp of qualitative research was chosen to meet the research aim. The study methodology – applying a realist ontological approach – was consistently applied throughout the whole research process.^30^ The study was conducted in Germany.

### Inclusion Criteria, Sampling, and Recruitment Strategy

Study inclusion was limited to state-recognized social workers working in the fields of general and family-oriented addiction counseling, and social pedagogical family support. Social workers needed work experience in one of these ambulatory settings for more than two years within the last three years to account for sufficient and current experience and expertise in the field.

A purposeful sampling strategy was applied to ensure the identification and selection of experienced social workers as interview partners.^31^ A heterogeneity of experts was targeted regarding the criteria of the exact working field and location to cover different working areas, states, and an urban and rural environment. Participation incentives were not given.

Key organizations and gatekeeping institutions working within general and family-oriented addiction support, as well as parenting counseling and social pedagogical family assistance, were contacted from July to September 2023. 15 professionals were initially contacted via phone or e-mail and requested for study participation after study information was provided. Six requested social workers did not participate in the study. The reason mentioned for that was too high workload due to shortage of staff at the workplace in all cases. The final study sample included nine expert interviews (Study Population Characteristics; see Table 1).

**Table 1:** Study Population Characteristics

Characteristic	Description	Number of Participants	Minimum/Maximum/Mean/Median
Sex	Female	9	
	Male	0	
Age	In years		28/64/45.89/45
Study entrance qualification	High school diploma	6	
	Childcare specialist with work experience	2	
	Advanced technical college certificate	1	
Name of study subject	Social work	5	
	Social pedagogy	2	
	Social welfare	1	
	Social work, Social pedagogy, Social welfare	1	
Highest academic qualification in social work or similar	Bachelor	5	
	Diploma	4	
Further job training	Addiction therapist	2	
	Systemic family therapist	2	
	Systemic supervisor	2	
	Childcare specialist with work experience	2	
	Childcare protection specialist	1	
	Creative writing	1	
	Nurse	1	
	Psychodrama	1	
Work experience	In years		4/40/18.4/19
State of workplace	Bavaria	2	
	Hessia	2	
	Hamburg	1	
	Mecklenburg-Western Pomerian	1	
	North Rhine-Westphalia	1	
	Saxony	1	
	Thuringia	1	
Working field	General addiction counseling	4	
	Social pedagogical family support	3	
	Family-oriented addiction counseling	2	

### Data Collection and Processing

The recommended principle by Helfferich was applied to create an interview guide. Thereby, potentially relevant interview questions were collected, checked, sorted, and finally subsumed.^32^ Based on current literature, findings on this topic, and a pre-test resulted in small adjustments and a rearrangement of the logical structure of the interview guide. The three main topics included social workers’ professional everyday experiences, perspectives and challenges in the context of inpatient rehabilitation, and, finally, their needs-oriented solution approaches in that field. The interviews were conducted in German via telephone or online video-call. The interviews were audio-recorded and fully transcribed verbatim by LM using the content-semantic transcription guideline by Dresing and Pehl.^33^ After the first transcription, all transcripts were audio rechecked, and mistakes were corrected accordingly. Pseudonymization was done to protect the participants and prevent their identification. After the interviews, the study participants filled out a short sociodemographic questionnaire; results are presented in Table 1.

### Data Analysis

Reflexive Thematic Analysis was selected as a suitable data analysis method, allowing for a flexible and deep interpretative analysis and iterative analytic corrections of the conducted interviews.^34–37^ Data collection and initial analysis were carried out by LM. LH, and AS provided continuous feedback as highly experienced qualitative researchers. Meetings were held to discuss the analytic process and the study results in a collaborative working method. Since an inductive research approach was applied and a set coding frame was not used, no inter-coder reliability was calculable in this Reflexive Thematic Analysis.^35^,^38^ The analysis of the interviews was conducted using the software NVivo (version 14.23.1 for Mac). Thereby, all six steps of Reflexive Thematic Analysis were conducted as initially outlined by Braun and Clarke.^34^,^36^ Firstly, the whole transcribed dataset was intensely read in the familiarization phase. Codes were then generated in a second step. This was done inductively, thus directly driven from the data without theoretical preconceptions^35^ and on a semantic level. Themes were then constructed by organizing codes. After extensively further exploring and interpreting the data, themes and subthemes were developed in a fourth step, using created thematic maps, and then named and defined in a fifth step. During the last step, the process of writing the report, analytical corrections were made again. The order of the theme presentation was decided on, and data extracts that illustrated the theme’s content were selected. The data collection was run in German, and so was the coding and theme development done in German to stay as close as possible to the original language and prevent early translation mistakes from distorting the analysis.

### Research Ethics and Data Protection

The criteria of the Code of Ethics of the German Sociological Association and the Professional Association of German Sociologists^39^ and the principles of the Declaration of Helsinki^40^ were applied during the whole research process. The overall study in which this study is embedded was approved by the Ethics Committee of the Technical University of Munich (reference number: 2022–624–S–KH). Prior to the start of the study, a data infrastructure concept was developed, which is in line with the German General Data Protection Regulation. All study documents (study information sheet, data protection sheet, and consent form) were handed out before the interview in German since the interview language was German. Participants agreed to their participation with their signature. The informed consent included the transcription, analysis, and publication of pseudonymized data in written form. They confirmed their informed and voluntary participation – after all questions regarding the study and the participation were answered – again just before the interview started.

### Quality Assurance

The Consolidated Criteria for Reporting Qualitative Research (COREQ) were used for considering quality standards throughout the research process. An additional quality criteria checklist with 15 items specialized in assessing the quality of the conducted Reflexive Thematic Analysis by Braun & Clarke was considered throughout study planning and conductance.^35^

## Results

### Participants’ Characteristics

Nine semi-structured expert interviews (mean duration: 58.2 minutes, minimum: 26.5 minutes, maximum: 84.8 minutes) were conducted in German via telephone (five interviews) and online video call (four interviews). All interview partners were state-recognized female social workers working in ambulatory contexts with parents with SUD and their children in Germany. Their working field was either general addiction counseling (four participants), family-oriented addiction counseling (two participants), or social pedagogical family support (three participants). The average age of the interview partners was 45.89 years (minimum: 28 years, maximum: 64 years), and the mean work experience in working with parents with SUD and their children was 18.4 years (minimum: 4 years, maximum: 40 years). More detailed study population characteristics can be found in Table 1.

### Themes

**Figure 1:**
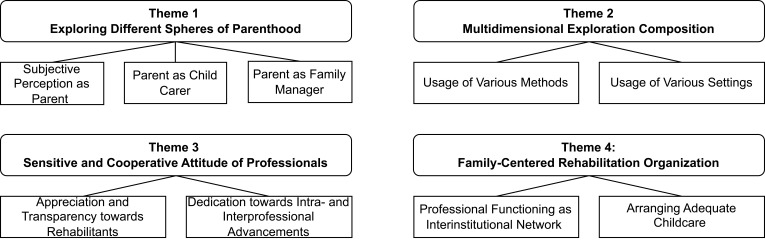
Thematic Map.

Four themes with each two to three subthemes were identified during data analysis to describe different aspects of how parents can be strengthened in the context of inpatient addiction rehabilitation (see overview Figure 1).

## Theme 1: Exploring Different Spheres of Parenthood

To strengthen parental rehabilitants in their parental role, the need for including an elaborate exploration of different spheres associated with parenthood into an intervention for parents in addiction rehabilitation was identified. These three exploration spheres for rehabilitants deal with the subjective perception as parent (subtheme 1.1), the parent as child carer (subtheme 1.2), and the parent as family manager (subtheme 1.3).

### Subtheme 1.1: Subjective Perception as Parent

By interviewees, the parental role was described as generally identity-forming for most of the clients. At the same time there are partially highly ambivalent emotional states going along with that role. This results in the need for an examination and discussion of the individual perception of themselves in their role as parents in a safe environment. Besides an increase in self-esteem, often feelings of insecurity, which can also be related to feelings of shame, guilt, anxiety, anger, frustration, grief, suppression of emotions and/or a feeling of overall disappointment in oneself, were reported. It is, therefore, crucial to explore how to deal with negatively perceived emotions constructively. Burdensome emotions were reported to be amplified by stigmatization processes produced by the social environment (by physicians, teachers, and state authorities like youth welfare offices), continuously questioning the clients’ ability to parent. As a result of internalized stigma and self-devaluation help-seeking can be deprived. One interview partner expressed the emotional spectrum going along with parenthood as follows:

I think having this parental role is always a big part of the person. And there lies a great potential in that. So, I can either experience a total boost in self-esteem perhaps through the role. But I can also experience total insecurity. [.] Because sometimes there is a lot of shame involved at first. Or maybe disappointment in yourself or frustration or anger. Or maybe suppression. A lot of grief. Whatever is involved [.] I believe that this must be prepared somehow. Access to your own role as a parent. And I believe that an exchange and working with the parental role is important.

It was additionally considered crucial to deal with the feelings, ideas, and wishes regarding one’s role as a parent, as one interview partner stated:

That they have an idea of what kind of person do I want to be when I stop drinking [.] How can I be? What might I actually be like? That would be really helpful, because then it is also easier to develop an attitude towards the children.

Becoming aware of what kind of parents rehabilitants want and can be and developing a positive but also realistic self-image and vision regarding that parental role is seen as helpful. Thereby, recognizing, exploring, and respecting personal boundaries can also be important for rehabilitants’ enhancing their self-confidence. Ultimately, positioning themselves toward their child(ren) and parent-child interactions can be improved.

### Subtheme 1.2: Parent as Child Carer

When considering significant interindividual differences in rehabilitants regarding their caring skills, it was identified as crucial to thematize child care in inpatient rehabilitation. Care includes everyday basic care tasks and child upbringing in that context. One participant pointed toward the deficits that need reduction in some parents regarding child handling:

Quite banal. I have to clean a toilet once a week so that the child can sit on it cleanly. A child needs a clean bottom, and it has to be dry before the diaper is put back on. [.] A child cries and you do not have to put a bread roll or a rusk or a cookie in their hand straight away. Many parents lack this very, very, very banal handling.

Here, the interview partner described the need for thematizing and acquiring essential knowledge and skills concerning the basic parental responsibilities required in everyday life. It is seen as necessary that parents understand the children’s basic physical and emotional needs and actions to meet these accordingly. Furthermore, often basic knowledge about the age-appropriate development of children, existing minimum legal standards regarding childcare (eg, in Germany participating in the 12 health-check-ups for children), potentially increased child support needs (occupational therapy, speech therapy, etc)., and the importance of recreational activities for children in and around the home setting (eg, going to the playground) and parental obligations connected to kindergarten and school are missing and need thematization during rehabilitation.

Further, discussing skillful parenting behavior and providing parenting advice was suggested by social workers to be included in an intervention. Through that, constructive and de-escalating ways of dealing with stressful parent-child interactions can be addressed. Preparing and supporting rehabilitants in dealing with their children was also identified as helpful, as sober parent-child interactions can be very different from the interaction perceptions and patterns to which they are used in everyday life under substance influence. An increased understanding of possible changes in child behavior after the inpatient rehabilitation stay compared to before and preparing them for that was also named as valuable. Within this context, developing an understanding of the children’s perspectives and a more sensitive understanding of children’s experiences (including potential traumatization of children) is stressed. Beyond that, it is important to thematize that good parenting behavior is not necessarily connected to constant conflict-free child-parent interactions. Instead, short-term conflicts, eg, because boundaries set by parents are maintained, can be normal and vital for a solid and consistent parent-child relationship. Making the parents aware that childcaring and upbringing are difficult can foster a realistic perception of the role of parents in childcaring and support them in finding their individual and suitable parenting style.

### Subtheme 1.3: Parent as Family Manager

Complex requirements regarding the planning and arrangement of everyday (family) life – subsumed in this context as family management – were often perceived as overwhelming. This is why it was suggested by social workers that rehabilitants are more intensely prepared during rehabilitation to cope with the complex obligations they face after the rehabilitation discharge regarding family life organization. These include everyday household tasks (eg, cleaning, shopping, cooking), a general increase of the appointment load, financial and bureaucratic obligations, and the reorganization of several life areas. Also, self-initiating the organization of help for personal support related to the SUD, childcare, or when generally overwhelmed with everyday responsibilities should be seen as a crucial skill to be thematized in the inpatient setting. Especially highly prevalent complex problem situations (see also thereby arising implications for interinstitutional cooperation in subtheme 4.1) can act as an immense stressor in everyday life:

Families are very often in very precarious social situations, right. Where this, let me put it this way, managing your own social life is something that overwhelms a lot of people. The fact that I have to submit a re-approval application for citizen’s allowance, that debts towards the health insurance fund/. Those are the classics. Debts towards the state justice fund. Most people want to move. Because when they take a sober look at their apartment from a distance, they say ‘that is not possible at all’. But then there is another organizational issue. And we have not even talked about the children yet, with school registration and daycare and lunch money providers and whatnot.

As shown here by one social worker, clients do not just have to manage practical everyday tasks but are – especially from an abstinent client perspective – confronted with several obligations like the processing of bureaucratic and financial tasks. All that results in a high workload, which can quickly be perceived as overwhelming.

Additionally, social workers highlighted the importance of parental communication about their SUD as a disease, its treatment, and planned actions in case of relapse within the family context. Interviewees described it as a common parental misbelief that children are not aware of their SUD. To learn how to communicate about their disease within the family context and particularly with their children – in an age-appropriate manner – is therefore crucial for honest interactions and rebuilding trust. Also, sharing the disease status with the environment (eg, in school and kindergarten) can bring relief and mobilize support options to improve family functioning.

## Theme 2: Multidimensional Exploration Composition

Within theme two, the need for using different methods when exploring the aforementioned parenthood-associated spheres (theme one) with the rehabilitants (subtheme 2.1) was identified. Additionally, the usage of a multitude of different social settings (subtheme 2.2) for the application of the different methods was found to be highly valuable within inpatient rehabilitation.

### Subtheme 2.1: Usage of Various Methods

Multiple social workers pointed towards the need for a great variation of applied methods – namely theoretical knowledge transfer, reflection, and practical exercises – when exploring different spheres associated with parenthood (subthemes 1.1–1.3).

Firstly, the need for theoretical input sessions to inform parents about basic knowledge eg, regarding basic children’s and teenagers’ needs and age-appropriate development, impacts of parental SUD on children, and different parenting styles, was stated throughout interviews.

Additionally, most interviewees stressed the importance of also involving (critical) self-reflection about personal family-related experiences, behaviors, and related thoughts and emotions eg, by using biography work and self-experience tasks, as one interviewed social worker stated:

So for me, the subject of self-experience is very important, yes. I usually come from addictive families, and I need to connect with my own history in order to then perhaps transfer it.

Another interviewee further explained:

So, and maybe to start there and say, okay, but what was it like for YOU before? What did YOU experience? What would you have wished for back then? And what would you have needed as a child, as a teenager?

It becomes clear in these statements that self-reflection is seen as crucial for getting in touch with one’s own history and experiences. For example, when rehabilitants remember their childhood, topics like personal needs and wishes as children can be examined and transferred to the potential needs and wishes of their children. Based on these reflections and the aforementioned knowledge transfer, constructive behaviors can be targeted for future parent-child interactions.

The importance of a practical exercise component for implementing more adequate ways of behaving towards the child was stated:

Perhaps also role-playing in the sense of social skills training. Maybe really practicing how to deal with difficult situations with children. Yes, because in the end, we are not fooling ourselves, so we can learn a lot about parenting in theory. When we get into a certain situation, I am still under stress and have to somehow apply what I have thought.

As described here by one social worker, examples of practical exercises can be role plays but also practicing how to handlestressful parent-child interactions. It was stressed that practicing is fundamental for acting appropriately in stressful situations and not falling into dysfunctional patterns.

### Subtheme 2.2: Usage of Various Settings

A multifaceted incorporation of different settings (single settings, one-to-one settings, and group settings) in which the different methods (subtheme 2.1) can be applied in an intervention was found to be central for strengthening parents in inpatient rehabilitation. Self-reflection tasks are mostly initially conducted alone (single setting), but insights and thoughts can also be shared later with others in a group setting or further discovered in a therapeutic one-to-one setting. The one-to-one setting can be used when concrete parental situations are professionally accompanied and analyzed to provide guidance for parents, as a social worker pointed out:

And that the parents are well supported when they realize, for example, how shitty it feels to interact with my child when you are not high. So that/. Or how nice it feels. At best, it is nice at some point, but we also experience the other option. And if this is well accompanied by social work or therapeutic discussions, from the parents’ perspective.

As it becomes visible here, one-to-one settings where professionals accompany parent-child interactions (which can be attributed to practical exercises) can be important for supporting rehabilitants in dealing with and reflecting on challenging situations and emotions. Some situations can be perceived as unfamiliar or challenging by the rehabilitants when they find themselves not being under the influence of a substance. The diversity of intense emotional states, which can arise during practical sessions, can influence the individual perception as a parent. These emotions should, therefore, be explored and discussed in a safe context in which this access is carefully and sensitively prepared and accompanied. In that way, the self-perception and the perception of professionals can both be included in consideration regarding training and educational advice.

Besides these one-to-one settings, group settings (as encounter or indicative groups) were mentioned as valuable intervention settings for theoretical knowledge transfer or to open the space for rehabilitants to exchange experiences with and challenges of their situation. A study participant verbalized:

[.] parents are really grateful for it [an indicative group for parents] because otherwise there is no room for it. […] And they also valued the opportunity to exchange ideas with each other, to experience with other parents that other parents have the same issues and the same problems as me. And also to be able to support each other with this or to be able to catch each other when parents perhaps want to get back in touch with children with whom they have had no or very little contact so far. And the children may not want that at all. So that, too, has to be dealt with somehow.

This statement reflects how valuable this peer setting can be perceived because, a unique feeling of trust and support can be developed. The understanding of other parents and tips for handling difficult situations, eg, when a child does not want to have contact with the parent, can go along with special credibility and emotional comfort.

## Theme 3: Sensitive and Cooperative Attitude of Professionals

For strengthening parents interview partners pointed towards the importance of the attitude of the health and social care professionals involved in the support provision. The required professional attitude was characterized by appreciation and transparency regarding rehabilitants (subtheme 3.1). as well as dedication regarding intra- and interprofessional advancements to also foster cooperation across professions and settings (subtheme 3.2).

### Subtheme 3.1: Appreciation and Transparency Towards Rehabilitants

Parents with SUD are often very cautious about discussing the topics of addiction and parenthood, fearing that their ability to care for and raise their children will be denied. Professionals described a sensitive “professional, humane attitude” as crucial so that rehabilitants feel taken seriously and safe. The professional attitude requires appreciation and transparency to allow establishing and maintaining a working relationship based on trust, respect, and dignity. This was seen as a prerequisite for constructive cooperation, including discussing critical topics. One social worker described it as follows:

I always take moms and dads seriously in this [parenting] role and address them specifically as experts for their children. [.] That doesn’t mean that you do not have a critical eye when a child’s welfare is at risk, but it is about the fundamental shaping of the relationship. And that is actually an old hat, is it not? That you first come into contact with each other through appreciation, and respect, and dignity that you nurture this little plant. And then, in the next step, if you have a relationship bonus, you can also work together quite well on more critical things, right?

The appreciative attitude described refers to the importance of approaching rehabilitants in their life situations non-judgmentally and with understanding. Also, showing continuous interest in rehabilitants, taking their fears seriously, praising their attempts and efforts, and encouraging them to shape their lives constructively.

Transparency refers primarily to a transparent way of communicating, especially regarding the topic of child welfare, child endangerment, and when and how related child protection measures must be initiated. Examining the well-being of the child critically does not exclude an attentive attitude toward the parents. Likewise, the goal of rehabilitation, which is an abstinent lifestyle, needs to be transparently communicated. Conversely, a constructive working relationship is damaged if parents feel disvalued, eg, when they feel ignored or patronized.

### Subtheme 3.2: Dedication Towards Intra- and Interprofessional Advancements

The aspect of intra-professional development includes sufficient professional expertise regarding the topics of addiction and family, eg, obtained by attending regular trainings and conferences. Ultimately, professionals should also be able to appropriately assess which tasks rehabilitants can and cannot realistically accomplish by themselves, hence, where and which professional support is currently needed. Furthermore, a constant (critical) examination of one’s own professional role(s) and the associated areas of expertise, the (powerful) position, and professional boundaries within the help system was pointed out.

Being aware of the professional responsibilities of all professions involved in the support system can also be understood as a professional ethical imperative. This enhances constructive cooperation across professions, institutions, and different settings. In addition, it is crucial for profound interprofessional cooperation that the professionals involved proactively initiate cooperative interactions, as one interview partner summarized:

There are cooperation agreements. […] However, in the end, these can only be filled if the individual professional deals with the issue and knows what they can and cannot do. And they are not afraid to call a specialist department and say, ‘I have this case and that case, and I do not know what to do. Can we talk about it?’

Just when individual professionals proactively initiate contact with other institutions and professionals, care for parents and children can be improved.

## Theme 4: Family-Centered Rehabilitation Organization

Interview partners stressed the importance of organizing the rehabilitation stay and follow-up care tailored to the family’s reality. Working as an interinstitutional cooperative network across settings is therefore considered highly necessary to sustainably strengthen the rehabilitants (subtheme 4.1). Since children are especially vulnerable, arranging adequate childcare and assuring their well-being need distinct attention (subtheme 4.2).

### Subtheme 4.1: Professional Functioning as Interinstitutional Network

Many interviewees mentioned the need for more intensive cooperation between different institutions of the support system across settings to ensure fast and needs-based care for rehabilitants. For better support, interface communication and interface coordination need to be improved on a system level between the various institutions and agencies that work (or should work) together in the family context. This relates to the time before, during, and after rehabilitation. The goal is to realize the seamless integration of support components and to locally provide continuous, suitable support services.

But everyone actually needs help afterwards to stabilize what they have learned there [in the rehabilitation clinic]. Because staying dry in such an inpatient setting, where there is a fixed daily routine with few stress points, is possible. But then to continue this at home, especially as parents [.]. It is difficult when the stress level rises, and everyone actually needs subsequent offers, which they do NOT all get reliably. So, or they have to wait a long time for it, and by the time they are ready, they have already relapsed and have to start all over again, it feels like.

Here, the social worker emphasized the necessity to provide immediate support for parents after rehabilitation discharge. After discharge, clients are again confronted with different challenges of everyday (family) life, and the likelihood of alcohol and/or drug consumption as a dysfunctional stress coping mechanism is high. This is why immediate support to strengthen and apply what was learned in everyday life situations is absolutely vital. A longer time without support can lead to a relapse, which diminishes the previous therapy successes. Confidentiality releases planned and implemented early in the support process were considered extremely important to improve interface communication and initiate reliable support services in a coordinated manner across settings. Communication between these institutions during the stay can be beneficial to exchange thoughts about the rehabilitation process (overall situation, progresses, problems) and the next steps to support the parent and children. Social pedagogical family assistance was seen as highly valuable after discharge. A weak institutional network was seen as a hurdle for interinstitutional cooperation around the rehabilitation stay. A good interinstitutional cooperation practice mentioned was the organization of regular neighborhood-based network meetings. In these, representatives of different institutions like kindergartens, schools, child and youth welfare associations, addiction support, and sometimes the police can be included. Another measure for improving cooperation was inviting representatives of other institutions to team meetings on an occasional basis. Building a well-functioning network structure takes time and effort, as one social worker described good interinstitutional cooperation as “[…] a really hard, long, hard road.”

### Subtheme 4.2: Arranging Adequate Childcare

An adequate care arrangement for the child during the rehabilitation stay is important to enable parental participation in inpatient rehabilitation and ultimately to ensure age-appropriate child development. If parents do not know that their children are well cared for, the likelihood is high for them not starting or early terminating the rehabilitation stay, as one interviewee formulated:

I often experience in conversations that it is like, ‘yes, that would be nice, BUT my children’. [.] ‘I cannot bear that my children are sad because I am not with them’. And that leads to thoughts of termination. [.] But also, yes, not being able to get involved because it is perhaps a bit of an uncertainty, ‘how well will the children be looked after now? […] Will I be able to get them back at all afterwards?’ These worries too […] That is something I often experience and hear. [.] So again, worries about what it will do to the child and the relationships back home, or about arriving afterwards? […] I think many parents are scared off by the fact that there is a lot of organization involved.

It becomes clear that the decision to (not) start with an inpatient rehabilitation is also heavily influenced by worries around childcare and anxiety that the respective child’s accommodation during the stay can have a negative impact on the parent-child relationship. The perceived uncertainties and fearing an organizational overload can cause great distress, partially leading to a decision not to enter rehabilitation. Hence, it is crucial to investigate the parents’ different options individually and support them in the needed coordination. Following an informed decision-making process, where the clients ideally make the final decision about the rehabilitation stay and childcare arrangements.

Besides the formal care arrangement, all interview partners expressed the need to strengthen children comprehensively and their resilience because the current support is generally perceived as insufficient. The reason for demanding more support offers is that growing up in a family with an affected parent can be highly challenging for children and be associated with risks for age-appropriate development. These needed separate support programs should be provided in an inpatient and/or a parallel outpatient setting that continues after the parental rehabilitation stay. The aim of all support services for children is to break the parental SUD pattern so that it is not intergenerationally transferred.

## Discussion

### Contextualization of the Results Within the Current State of Research

Our analysis identified four essential themes, that social workers regard as essential for strengthening parents with SUD in inpatient addiction rehabilitation. When developing an intervention for rehabilitants these key components should be carefully considered and implemented. Firstly, an intervention should facilitate an exploration of rehabilitants’ perceptions and roles as parents. Secondly, in doing so, it is important to employ diverse methods and settings to enhance learnings and reflection. Thirdly, the importance of a supportive professional attitude characterized by appreciation and transparency of professionals involved in the care should be emphasized. Lastly, the intervention should include a family-centered approach by encouraging interinstitutional collaboration and ensuring adequate childcare support during and after rehabilitation.

In line with former studies, our results show that the subjective perception of rehabilitants as parents can be ambivalently described and strongly negatively influenced by stigmatization processes, which disapprove adequate parenthood of parents with SUD. Also, when (expected) motherhood can become a positive motivation for recovery,^41^,^42^ the societal reproduced expectations of how to “be a good mother” can also be experienced as burdensome^43^ and impact the recovery trajectory.^44^ Therefore, strengthening parents to face and distance themselves from prejudices and stigmatization and pacifying inner conflicts related to their parental role and their SUD could become a facilitator for increasing rehabilitants’ confidence in their parental role. These reflections should also include, as Sieger and Haswell^45^ conclude in their study, the negative (harmful) but also positive (helpful) attributes parents with SUD associate with the SUD. As suggested in our results, dealing with the feelings, ideas, and wishes regarding one’s parental role could contribute to the respective parental identity transition and foster recovery trajectories. According to Neger and Prinz,^19^ reflection in parents should first focus on their persona and emotional regulation before childcare is focused. This recommendation is in line with the evaluation of the multi-component Enhancing Permanency in Children and Families program (EPIC) for opioid-dependent parents conducted in the US.^46^ The substance-misusing participants did value the intervention component focusing on parenting skills within the Nurturing Parenting Program to enhance relational skills the least. That could indicate – as the authors state – that the participants of this study prioritized achieving abstinence and support components more directly linked to that. Nevertheless, this could also be due to the program design and delivery potentially focusing too little on subjective parenting experiences and emotions through which the understanding of the importance of dealing with parenthood and parenting skills could be increased. According to attachment theory, including parenting skills and the influence of relational and emotional parent-child bond can be beneficial, as Suchman et al^15^ as well as Parolin and Simonelli,^47^ summarize. Empirical findings show the link between (insecure) parent-child attachment, eg, based in parental neglect,^48^ and SUD etiology.^49–53^ This is why a basic understanding of attachment theory and patterns can be highly valuable for rehabilitants to understand their own SUD development as well as their potentially severe impact on their child’s development.^54^ Nevertheless, for parenting interventions in SUD treatment, it is important to target the rehabilitants’ own childhood experiences from becoming and being parents distinctly.^55^ Wiegand-Grefe et al^56^ and the results of our study stress the need for parents to become aware of how SUD changes their perception and behavior, also regarding their children and the children’s needs and their satisfaction. This is why adequate childcare and handling are recommended to be addressed in an intervention, even though the order and the methodological approach for doing so are not without contradictions and need further research.

Additionally, introducing and implementing sustainably meaningful family rituals and reliable daily routines (eg, having dinner together), by fostering parents as family managers, can improve well-being in adults and children.^57^,^58^ Besides bad housekeeping, insufficient communication is often prevalent in families with parents with SUD, as Geene and Böhm^59^ summarize in line with our results. Our study results show that open communication with the children about the taboo topic of parental SUD can be a great relief and resilience factor that can improve overall family interactions. This is in line with other research results.^60–62^ Zeng and Tan^63^ even underlined that good communication is part of good family functioning, which is again negatively correlated with relapse tendencies.

The intervention components mentalizing stance, mentalizing for the mother, and mentalizing for the child are part of the evidenced-based intervention Mothering from the Inside Out (MIO) for mothers with SUD.^14^ These can be linked to the usage of methods to reflect on the own and the child’s perspectives suggested in our results. In our results, sharing self-reflection insides with others, like professionals and/or former rehabilitants, was additionally found to be valuable. Especially in peer support settings, a sense of belonging can be created, which can become a mediator for better recovery, as Bliuc et al^64^ state. In the multicomponent program evaluation of the program,^46^ support by trained ex-users was a component highly valued by patients. Examinations of peer-to-peer support point toward the need for great collaborative efforts, eg, regarding matching current and former rehabilitants, task and role definition, compliance, and supervision regulations, between all stakeholders to incorporate stable peer-to-peer support in regular services. These complex hurdles for a sustainable implementation of peer-to-peer support must be considered before deciding to incorporate peer-to-peer services; otherwise, a high turnover and instability in peer-to-peer support can be caused.^46^,^65^,^66^

The practical exercises recommended in our study bear the potential to open a room to practice how parenting goals can be achieved in a (near) real-life situation, eg, by simply accompanying (also conflictual) parent-child interactions and supervised reflections, as also Wiig et al^67^ point toward to. Especially when entering the support system, which confronts parents with SUD with unknown contexts and situations, patients can feel powerless, dependent on the mercy of others, and insecure about what to expect.^68^ (Mis)trust within the working relationship can significantly impact the success or failure of the support offered.^69^ In family-oriented addiction support, an open professional attitude is the basis for cognitive, affective, and action-related interactions, which massively influence the relationship between the professional and the patient.^70^ Also, in several other studies,^46^,^67^,^69^,^71^ a trustworthy and supportive experienced relationship between patients and staff was described as extremely vital for patient’s treatment engagement. This is in line with the results of our study, which highlight that an appreciative attitude and a trustworthy working relationship are crucial for rehabilitation. In ambulatory family-oriented addiction support, the need for professionals to be aware of their specific tasks, work assignments, and methods is already emphasized.^23^ Treatment priorities can, eg, based on different professionals’ work experience, differ even within disciplines, potentially leading to tensions.^67^ For good interprofessional cooperation (including good information exchange), it is, as shown in our results, vital that the professionals respectfully, appreciatively, and transparently work at eye level with each other. Generally, literature focuses more on the attitude of professionals in terms of the professional-patient relationship and less in terms of the attitude of professionals towards each other. Our study stresses the importance of engaged professionals as the basis of interinstitutional cooperation, described hereinafter.

Like our results, Wiegand-Grefe et al^72^ outline that many families of parents with SUD have already had contact with many different players of the social security system, often based on precarious living circumstances (including poverty and educational disadvantages) characterized by multi-problem situations. Hence, it is important to focus on the rehabilitants, including the respective influencing contextual factors.^73–75^ Especially, a better connection between the addiction support system and child and youth welfare, eg, via a routine integration of social pedagogical family assistance in the follow-up care of parents with SUD, was stressed in our study. Aligning with our results, other studies^22^ and projects focusing on parents with SUD in Germany^76^ and abroad conclude that cross-agency cooperation^46^,^77^,^78^ is crucial in clinical settings. Nevertheless, there still is a lack of sufficient supply of support services and a lack of the implementation of functional cooperation structures between the help systems, leading to interface problems, which have been known for decades.^23^ Hansjürgens^79^ also points out – in line with our results – that better interinstitutional and inter-setting cooperation could prevent the so-called “revolving door effect” (a repeated admission to an inpatient setting shortly after discharge) or early and permanent exclusion from the support system. Better communal cooperation is also a key element of better care and prevention for children and teenagers of parents with SUD.^6^ Planning the rehabilitation stay in line with the family reality is also crucial when it comes to the organization of adequate childcare arrangements in the context of rehabilitation. A Canadian focus group discussion by Elms et al^80^ found that for mothers, one barrier to accessing SUD treatment was a lack of proper childcare, respectively a fear of insufficient welfare of the child, and a fear of losing child custody. This is in line with our findings stressing the need for appropriate childcare to enable inpatient rehabilitation for parents. Also, our results stressed the importance of strengthening not just the parents but also fostering protective factors for children’s development and their resilience, as also Velleman and Templeton^81^ point towards to.

### Implications for Research

Social workers see high potential in strengthening parents in inpatient addiction rehabilitation. The implementation and scientific evaluation of interventions, including the context factors of parenthood and family in inpatient addiction rehabilitation, is therefore promising. Further research should additionally consider the following.

Firstly, most research on parents with SUD is conducted on mothers,^82^ which is why more research is needed on fathers as well. Considering traditional implications of the roles of mothers and fathers – including societal expectations and associated stigmatization if these are not fulfilled – it should be further explored if and what kind of gender-sensitive components should be included to optimally meet gender-specific needs of parents in the context of rehabilitation. Additionally, future study samples should be more diverse regarding the cultural backgrounds for a better culture-sensitive intervention composition.

Secondly, some interviewees mentioned that professionals should not just account for building an excellent professional formal network around the rehabilitants. Rather, they should also strengthen the informal care network of rehabilitants. This includes friends, family members, neighbors, other community members, and volunteers. Research in the field of community support shows promising results for further strengthening parents in rehabilitation and recovery.^44^,^83^ It seems to be valuable to explore more in-depth how informal networks could be utilized.

Thirdly, it can be rewarding to examine further the factors that influence the professional attitude of professionals involved in the care. Thereby, especially the attitude towards intraprofessional reflection and interprofessional cooperation should be inspected since both play a fundamental role in stabilizing families. In this context, it is recommended to include not only perspectives of social workers but of other professionals involved in the care as well. Targeting improvements in care provision and interprofessional collaboration comparatively analyzing similarities and divergences of different professional logics and perspectives – influencing attitudes and practices – can be highly important.

Fourthly, more research is needed on formal success components regarding the concrete implementation of the intervention. The intervention content to be incorporated in the intervention and methods of conveying the respective content can be concluded from theme one and two, which can directly be used to improve current policies. Nevertheless, the exact order of intervention modules and practical training sessions remains to be explored more in depth in rehabilitation practise.

### Study Limitations and Strength

Regardless of careful study preparation and conductance, certain limitations need to be acknowledged hereinafter. No male interview partners were interviewed. This sex-related homogeneity, not allowing for an analysis of men’s perspectives, was not intended, but is also not surprising, considering the much higher proportion of women in social work and family educational support services. The male social workers who were requested to participate in the study decided against participating. The reason mentioned was a too high workload due to a shortage of staff at the workplace in all cases.

A purposeful sampling strategy was carefully applied, and even though sampling was not iterative, a great range of variation was reached in other areas than participants’ sex. Interviewed social workers’ perspectives on how parents can be strengthened in their parental role are generally limited to their professional ambulatory standpoint. Nevertheless, this examined perspective of social workers in ambulatory addiction support and addiction-related family support is very valuable since they work the closest to everyday life settings of parents with SUD and provide crucial insights into actual living realities. When planning and implementing an intervention to strengthen parents with SUD in inpatient rehabilitation the perspective of other professions and work settings is a crucial addition to these study findings.

Despite these critical considerations regarding study shortcomings, the conducted study includes several strengths. The study subject is highly topical, and the study population was motivated to participate, which becomes visible in the relatively high participation ratio, reflecting the willingness to share their insights. The pre-tested and slightly adapted semi-structured interview guide afterward proved well implementable and manageable in the research context while leaving space for spontaneous narratives and emphasis on the specific contents of interviewees. The research methodology was chosen carefully and appropriately for the research interest, and measures to ensure a high quality of the whole research project were taken, leading to information-rich data.

## Conclusion

SUD causes a substantial burden on a societal^10^ and an individual level for the affected parents, but also their social surroundings and especially the healthy development of their children.^6^ Considering the great responsibility load of parenthood and the stress going along with that, parents with a mental disease like SUD can be quickly overwhelmed and need support in fulfilling their parental role adequately.^23^ Inpatient rehabilitation is potentially a promising context in which parents can be strengthened in their parental role, but parenthood and family-associated interventions are in the clinical setting not routinely and evidence-based implemented in Germany.

Nine semi-structured interviews were conducted with social workers highly involved in supporting parents with SUD and their children. The interviews aimed to find out how parents can be strengthened in the context of inpatient addiction rehabilitation and were analyzed using a Reflexive Thematic Analysis.^34–36^ The results show, that the thematization of parenthood ought to cover an in-depth exploration of different parenthood-associated spheres, namely their subjective perception as a parent, their role as a child carer, and as a family manager. A pluralism of applied methods (theoretical knowledge transfer, reflection, and practical exercise components) and settings (single setting, one-to-one setting, group setting) is essential for that multifaceted examination. The attitude of professionals towards the rehabilitants – ideally characterized by appreciation and transparency – as well as towards other professionals is important for the success of treatment impulses. Furthermore, the sustainability of treatment successes depends on coordinated cooperation between institutions and services before, during, and after an inpatient rehabilitation stay. Following these suggestions and aligning them to the respective overall therapeutic concept of a rehabilitation clinic and the regionally differing cooperation partners can be seen as promising to strengthen parents hence their children, and the family, and decrease the overall disease burden also on a societal level. Depending on the societal perception of and discourse about addiction and the political framework,^84^ addiction support changes over time, and different missions and mandates of institutions are formed.^85^ In an international comparison, the support system for persons with SUD is relatively well-equipped in Germany.^86^ Nevertheless, care provision is nationwide very heterogeneous.^22^ That is especially problematic for persons in need of support living in regions with few support options. Political decisions – which influence the structure and funding frame of care offered^68^ – should foster holistic care provision that ensures adequate care in all regions. Furthermore, more financial means should be provided to promote innovative models of care to explore treatment and rehabilitation options with sustainable long-term effects for meeting existing needs fostering structural improvements. For that, it is crucial to evaluate the effectiveness of an intervention design incorporating the identified recommendations of social workers applying a quantitative study design.

## Abbreviations

SUD, substance use disorder; WHO, World Health Organization.

## References

[1] Global Status Report on Alcohol and Health and Treatment of Substance Use Disorders. World Health Organization; 2024.

[2] Laging M. *Soziale Arbeit in Der Suchthilfe: Grundlagen Konzepte – Methoden. 1*. Kohlhammer; 2018.

[3] Kuppens S, Moore SC, Gross V, Lowthian E, Siddaway AP. The enduring effects of parental alcohol, tobacco, and drug use on child well-being: a multilevel meta-analysis. *Dev Psychopathol*. 2020;32(2):765–778. doi:10.1017/S0954579419000749 31274064 PMC7525110

[4] Lipari RN, Van Horn SL. Children living with parents who have a substance use disorder. In: *Center for Behavioral Health Statistics and Quality Substance Abuse and Mental Health Services Administration (SAMHSA)*. 2017:1–7. 29144715

[5] Kraus L, Uhl A, Atzendorf J, Seitz NN. Estimating the number of children in households with substance use disorders in Germany. *Child Adolesc Psychiatr Ment Health*. 2021;15(1):63. doi:10.1186/s13034-021-00415-0 PMC8571854 34740375

[6] Thomasius R, Klein M. *Literatur- Und Datenbankrecherche Zu Gesundheitsförderungs- Und Präventionsansätzen Bei Kindern Aus Suchtbelasteten Familien*. Ergebnisbericht.; 2018.

[7] Klein M, Thomasius R, Moesgen D. *Kinder von Suchtkranken Eltern – grundsatzpapier Zu Fakten Und Forschungslage*. Die Drogenbeauftragte der Bundesregierung; 2017. Available from: https://www.bundesgesundheitsministerium.de/fileadmin/Dateien/5_Publikationen/Drogen_und_Sucht/Broschueren/Broschuere_Kinder_aus_suchtbelasteten_Familen.pdf. Accessed June 29, 2023.

[8] Wilkens K. *Kinder und Jugendliche aus suchtbelasteten Familien als Herausforderung der Kinder- und Jugendhilfe. „Wie findet die Alkoholerkrankung eines oder beider Elternteile Berücksichtigung im Umgang mit problembelasteten Kindern und Jugendlichen auf Seiten der Kinder- und Jugendhilfe?”*. BIS-Verlag der Carl von Ossietzky Universität Oldenburg; 2017.

[9] Maurach LM, Wolstein J. Identitätsentwicklung und Bewältigung weiterer Entwicklungsaufgaben bei Kindern alkoholkranker Eltern im Übergang zum Erwachsenenalter. *SUCHT*. 2019;65(3):161–174. doi:10.1024/0939-5911/a000603

[10] Effertz T. Kosten bei Kindern aus Suchtfamilien: die volkswirtschaftliche Dimension eines kaum beachteten Problems. Available from: https://nacoa.de/sites/default/files/images/stories/pdfs/vortrag%20effertz%20volkswirtschafliche%20kosten.pdf. Accessed September 04, 2025.

[11] Moesgen D, Schulz W, Klein M. Elterliche Alkoholprobleme: kognitionen der Kinder und Verhaltensauffälligkeiten. *SUCHT*. 2012;58(2):109–118. doi:10.1024/0939-5911.a000170

[12] DRB DRV. *Entwöhnungsbehandlung – Ein Weg aus der Abhängigkeit*. Deutsche Rentenversicherung Bund Abteilung Presse- und Öffentlichkeitsarbeit, Kommunikation; 2022.

[13] Sperlich MI, Bascug EW, Green SA, Koury S, Hales T, Nochajski TH. Trauma-informed parenting education support groups for mothers in substance abuse recovery. *Res Soc Work Pract*. 2021;31(7):742–757. doi:10.1177/10497315211007568

[14] Lowell AF, Peacock-Chambers E, Zayde A, DeCoste CL, McMahon TJ, Suchman NE. Mothering from the inside out: addressing the intersection of addiction, adversity, and attachment with evidence-based parenting intervention. *Curr Addict Rep*. 2021;8(4):605–615. doi:10.1007/s40429-021-00389-1 34306964 PMC8280593

[15] Suchman N, Mayes L, Conti J, Slade A, Rounsaville B. Rethinking parenting interventions for drug-dependent mothers: from behavior management to fostering emotional bonds. *J Subst Abuse Treat*. 2004;27(3):179–185. doi:10.1016/j.jsat.2004.06.008 15501370

[16] Mazel S, Alexander K, Cioffi C, Terplan M. Interventions to support engagement in addiction care postpartum: principles and pitfalls. *Subst Abuse Rehabil*. 2023;14:49–59. doi:10.2147/SAR.S375652 37424702 PMC10327918

[17] Choi S, Rosenbloom D, Stein MD, Raifman J, Clark JA. Differential gateways, facilitators, and barriers to substance use disorder treatment for pregnant women and mothers: a scoping systematic review. *J Addict Med*. 2022;16(3):e185–e196. doi:10.1097/ADM.0000000000000909 34380985 PMC8828806

[18] Petzold J, Spreer M, Krüger M, et al. Integrated care for pregnant women and parents with methamphetamine-related mental disorders. *Front Psychiatry*. 2021;12:762041. doi:10.3389/fpsyt.2021.762041 34759851 PMC8573098

[19] Neger EN, Prinz RJ. Interventions to address parenting and parental substance abuse: conceptual and methodological considerations. *Clin Psychol Rev*. 2015;39:71–82. doi:10.1016/j.cpr.2015.04.004 25939033 PMC4464898

[20] Dyba J, Moesgen D, Klein M, Leyendecker B. Mothers and fathers in treatment for methamphetamine addiction—Parenting, parental stress, and children at risk. *Child Fam Soc Work*. 2019;24(1):106–114. doi:10.1111/cfs.12587

[21] Rutherford H, Mayes LC. Parenting stress: a novel mechanism of addiction vulnerability. *Neurobiol Stress*. 2019;11:100172. doi:10.1016/j.ynstr.2019.100172 31193862 PMC6543178

[22] Helsper N, Kemner K, Arnold J, Feist-Ortmanns M. *Steuerungswissen Und Handlungsorientierung Für Den Aufbau Effektiver Interdisziplinärer Versorgungsnetzwerke Für Suchtbelastete Familien - Vorläufiger Abschlussbericht*; 2022. Available from: https://jugendhilfe-suchthilfe.de/wp-content/uploads/sites/4/2022/04/Vorl%C3%A4ufiger-Abschlussbericht-Steuerungswissen-und-Handlungsorientierung_Versorgung-suchtbelasteter-Familien.pdf. Accessed January 22, 2024.

[23] 2021. Available from: https://www.eltern-sucht.de/wp-content/uploads/2021/08/rahmenkonzept_suchtarbeit_bf_end.pdf. Accessed September 04, 2025.

[24] Stullich A, Hoffmann L, Stephan J, Gehrmann J, Richter M. Evaluating a rehabilitative intervention for substance-dependent patients with and without their accompanying children in Germany (KontextSucht): study protocol for a non-randomised trial. *BMJ Open*. 2024;14(3):e078148. doi:10.1136/bmjopen-2023-078148 PMC10941178 38485489

[25] rehapro. Projektdarstellung KontextSucht. Available from: https://www.modellvorhaben-rehapro.de/SharedDocs/Projektdaten/Projektdarstellung_KontextSucht_2FA.html. Accessed January 24, 2024.

[26] Blankenburg K, Cosanne E. Gesellschaftliche Trends und Beschäftigtenzahlen in Praxisfeldern gesundheitsbezogener Sozialer Arbeit. In: Dettmers S, Bischkopf J, editors. *Handbuch gesundheitsbezogene Soziale Arbeit*. 2nd ed. Ernst Reinhardt Verlag; 2019:138–146.

[27] Meyer C, Oelkers N. Soziale Arbeit. mit Familien. In: Graßhoff G, Renker A, Schröer W editors. *Soziale Arbeit: Eine elementare Einführung*. Lehrbuch. Springer VS; 2018:151–168. doi:10.1007/978-3-658-15666-4.

[28] Oswald C, Meeß J. *Methodenhandbuch Kinder und Jugendliche aus suchtbelasteten Familien*. Lambertus; 2019.

[29] Laging M. *Soziale Arbeit in der Suchthilfe: Grundlagen - Konzepte - Methoden. 3. überarbeitete Auflage*. Verlag W. Kohlhammer; 2023.

[30] Przyborski A, Wohlrab-Sahr M. *Qualitative Sozialforschung: Ein Arbeitsbuch*. 4. erweiterte Auflage ed. Oldenbourg Verlag; 2014.

[31] Patton MQ. *Qualitative Research & Evaluation Methods*. 3 ed. [Nachdr.]. Sage; 2010.

[32] Helfferich C. *Die Qualität qualitativer Daten: manual für die Durchführung qualitativer Interviews*. Auflage VS Verl für Sozialwiss. 2011.

[33] Dresing T, Pehl T. *Praxisbuch Interview, Transkription & Analyse: Anleitungen und Regelsysteme für qualitativ Forschende*. 8th ed. Eigenverlag; 2018.

[34] Braun V, Clarke V. Using thematic analysis in psychology. *Qual Res Psychol*. 2006;3(2):77–101. doi:10.1191/1478088706qp063oa

[35] Braun V, Clarke V. *Successful Qualitative Research: A Practical Guide for Beginners*. SAGE; 2013.

[36] Braun V, Clarke V. *Thematic Analysis: A Practical Guide*. SAGE; 2022.

[37] Terry G, Hayfield N. *Essentials of Thematic Analysis*. American Psychological Association; 2021; doi:10.1037/0000238-000

[38] O’Connor C, Joffe H. Intercoder reliability in qualitative research: debates and practical guidelines. *Int J Qual Methods*. 2020;19:1609406919899220. doi:10.1177/1609406919899220

[39] DGS (Deutschen Gesellschaft für Soziologie). BDS (Berufsverbandes Deutscher Soziologinnen und Soziologen). Ethik-Kodex der Deutschen Gesellschaft für Soziologie (DGS) und des Berufsverbandes Deutscher Soziologinnen und Soziologen (BDS). Available from: https://soziologie.de/fileadmin/user_upload/dokumente/Ethik-Kodex_2017-06-10.pdf. Accessed January 25, 2024.

[40] World Medial Association. WMA declaration of Helsinki - ethical principles for medical research involving human subjects. Available from: https://www.wma.net/policies-post/wma-declaration-of-helsinki-ethical-principles-for-medical-research-involving-human-subjects/. Accessed September 04, 2025.

[41] Neale J, Sheard L, Tompkins CN. Factors that help injecting drug users to access and benefit from services: a qualitative study. *Subst Abuse Treat Prev Policy*. 2007;2(1):31. doi:10.1186/1747-597X-2-31 17971204 PMC2169215

[42] Weber A, Miskle B, Lynch A, Arndt S, Acion L. Substance use in pregnancy: identifying stigma and improving care. *Subst Abuse Rehabil*. 2021;12:105–121. doi:10.2147/SAR.S319180 34849047 PMC8627324

[43] Adams ZM, Ginapp CM, Price CR, et al. “A good mother”: impact of motherhood identity on women’s substance use and engagement in treatment across the lifespan. *J Subst Abuse Treat*. 2021;130:108474. doi:10.1016/j.jsat.2021.108474 34118710 PMC8478714

[44] Collinson B, Hall L. The role of social mechanisms of change in women’s addiction recovery trajectories. *Drugs Educ Prev Policy*. 2021;28(5):426–436. doi:10.1080/09687637.2021.1929077

[45] Sieger MHL, Haswell R. Family treatment court-involved parents’ perceptions of their substance use and parenting. *J Child Fam Stud*. 2020;29(10):2811–2823. doi:10.1007/s10826-020-01743-z

[46] Shockley McCarthy K, Price Wolf J, Dellor E. Promoting permanency in families with parental substance misuse: lessons from a process evaluation of a multi-system program. *BMC Public Health*. 2022;22(1):2261. doi:10.1186/s12889-022-14528-4 36463173 PMC9719642

[47] Parolin M, Simonelli A. Attachment theory and maternal drug addiction: the contribution to parenting interventions. *Front Psychiatry*. 2016;7. doi:10.3389/fpsyt.2016.00152 PMC5004230 27625612

[48] Dunn MG, Tarter RE, Mezzich AC, Vanyukov M, Kirisci L, Kirillova G. Origins and consequences of child neglect in substance abuse families. *Clin Psychol Rev*. 2002;22(7):1063–1090. doi:10.1016/S0272-7358(02)00132-0 12238246

[49] Zhai ZW. *The Role of Attachment to Parents in the Etiology of Substance Use Disorder*. University of Pittsburgh; 2015.

[50] Schindler A. Attachment and substance use disorders—theoretical models, empirical evidence, and implications for treatment. *Front Psychiatry*. 2019;10:727. doi:10.3389/fpsyt.2019.00727 31681039 PMC6803532

[51] Guyon-Harris KL, Jacobs J, Lavin K, Moran Vozar TE. Perinatal substance use and the underpinnings of addiction and attachment: implications for parenting interventions. *Pract Innov*. 2023;8(2):75–88. doi:10.1037/pri0000199

[52] Gerra ML, Gerra MC, Tadonio L, et al. Early parent-child interactions and substance use disorder: an attachment perspective on a biopsychosocial entanglement. *Neurosci Biobehav Rev*. 2021;131:560–580. doi:10.1016/j.neubiorev.2021.09.052 34606823

[53] Lander L, Howsare J, Byrne M. The impact of substance use disorders on families and children: from theory to practice. *Soc Work Public Health*. 2013;28(3–4):194–205. doi:10.1080/19371918.2013.759005 23731414 PMC3725219

[54] Cocio-Thompson J. *Improving Attachment and Bonding: Infants Born with Neonatal Abstinence Syndrome*. Northeastern University; 2025.

[55] Flykt M, Belt R, Salo S, Pajulo M, Punamäki RL. Prenatal reflective functioning as a predictor of substance-using mothers’ treatment outcome: comparing results from two different RF measures. *Front Psychol*. 2022;13:909414. doi:10.3389/fpsyg.2022.909414 35959038 PMC9359121

[56] Wiegand-Grefe S, Klein M, Kölch M, et al. *Kinder psychisch kranker Eltern „Forschung”. IST-Analyse zur Situation von Kindern psychisch kranker Eltern*; 2019. Available from: https://www.ag-kpke.de/wp-content/uploads/2019/02/Stand-der-Forschung-1.pdf. Accessed September 04, 2025.

[57] Zobel M. *Kinder Aus Alkoholbelasteten Familien*. 3rd. Hogrefe; 2017. doi:10.1026/02830-000

[58] Weisner TS. Well-being, chaos, and culture: sustaining a meaningful daily routine. In: Evans GW, Wachs TD editors. *Chaos and Its Influence on Children’s Development: An Ecological Perspective*. American Psychological Association; 2010:211–224. doi:10.1037/12057-013.

[59] Geene R, Böhm K. Kinder aus suchtbelasteten Familien – lebenssituation und Unterstützungsbedarf. In: Marchwacka MA editor. *Gesundheitsförderung im Setting Schule*. Springer Fachmedien Wiesbaden; 2013:83–96. doi:10.1007/978-3-658-00528-3.

[60] Lenz A, Wiegand-Grefe S. *Kinder psychisch kranker Eltern. 1*. Auflage. Hogrefe; 2017.

[61] Velleman RDB, Templeton LJ, Copello AG. The role of the family in preventing and intervening with substance use and misuse: a comprehensive review of family interventions, with a focus on young people. *Drug Alcohol Rev*. 2005;24(2):93–109. doi:10.1080/09595230500167478 16076580

[62] Hemati Z, Abbasi S, Oujian P, Kiani D. Relationship between parental communication patterns and self-efficacy in adolescents with parental substance abuse. *Iran J Child Neurol*. 2020;14(1):49–56. 32021628 PMC6956959

[63] Zeng X, Tan C. The relationship between the family functioning of individuals with drug addiction and relapse tendency: a moderated mediation model. *Int J Environ Res Public Health*. 2021;18(2):625. doi:10.3390/ijerph18020625 33451020 PMC7828550

[64] Bliuc AM, Best D, Moustafa AA. Accessing addiction recovery capital via online and offline channels: the role of peer-support and shared experiences of addiction. In: Moustafa AA, editor. *Cognitive, Clinical, and Neural Aspects of Drug Addiction*. Academic Press; 2020:251–266.

[65] Fallin-Bennett A, Elswick A, Ashford K. Peer support specialists and perinatal opioid use disorder: someone that’s been there, lived it, seen it. *Addict Behav*. 2020;102:106204. doi:10.1016/j.addbeh.2019.106204 31794901

[66] Olding M, Cook A, Austin T, Boyd J. “They went down that road, and they get it”: a qualitative study of peer support worker roles within perinatal substance use programs. *J Subst Abuse Treat*. 2022;132:108578. doi:10.1016/j.jsat.2021.108578 34373170

[67] Wiig EM, Halsa A, Bramness J, Myra SM, Haugland BSM. Rescue the child or treat the adult? Understandings among professionals in dual treatment of substance-use disorders and parenting. *Nord Stud Alcohol Drugs*. 2018;35(3):179–195. doi:10.1177/1455072518773615 PMC7434155 32934526

[68] Tretter F. *Sucht. Gehirn. Gesellschaft*. Medizinisch Wissenschaftliche Verlagsgesellschaft; 2017.

[69] Wangensteen T, Hystad J. Trust and collaboration between patients and staff in SUD treatment: a qualitative study of patients’ reflections on inpatient SUD treatment four years after discharge. *Nord Stud Alcohol Drugs*. 2022;39(4):418–436. doi:10.1177/14550725221082366 PMC9379296 36003119

[70] Albrecht R. Beratungskompetenz in der Sozialen Arbeit: auf die Haltung kommt es an! *Kontext*. 2017;48(1):45–64. doi:10.13109/kont.2017.48.1.45

[71] Fowler C, Reid S, Minnis J, Day C. Experiences of mothers with substance dependence: informing the development of parenting support. *J Clin Nurs*. 2014;23(19–20):2835–2843. doi:10.1111/jocn.12560 25280136

[72] Wiegand-Grefe S, Mattejat F, Lenz A. *Kinder mit psychisch kranken Eltern: Klinik und Forschung: Mit 55 Tabellen*. Vandenhoeck & Ruprecht; 2011.

[73] Matting A. Eine psychologische Sicht. *SozialAktuell*. 2016;48(12):10–11.

[74] Suchman NE, Luthar SS. Maternal addiction, child maladjustment and socio‐demographic risks: implications for parenting behaviors. *Addiction*. 2000;95(9):1417–1428. doi:10.1046/j.1360-0443.2000.959141711.x 11048359 PMC1852451

[75] Behrendt K, Backmund M, Reimer J. *Drogenabhängigkeit*. 6th ed. Deutsche Hauptstelle für Suchtfragen e. V;2021.

[76] Stürmer M. [Schulterschluss] Für Kinder & Jugendliche in Suchtbelasteten Familien. Impulse Aus Dem Bayernweiten Kooperationsprojekt. *Abschlussbericht Projektphasen I+II*. 2019. https://www.schulterschluss-bayern.de/fileadmin/user_upload/schulterschluss_Abschlussbericht-2-2019.pdf. Accessed September 4, 2025.

[77] John McConnell K, Kaufman MR, Grunditz JI, et al. Project nurture integrates care and services to improve outcomes for opioid-dependent mothers and their children: study assesses project nurture, an innovative model that integrates maternity care, substance use treatment, and social service coordination for medicaid beneficiaries in Portland, Oregon. *Health Aff*. 2020;39(4):595–602. doi:10.1377/hlthaff.2019.01574 32250679

[78] Schiff DM, Partridge S, Gummadi NH, et al. Caring for families impacted by opioid use: a qualitative analysis of integrated program designs. *Acad Pediatr*. 2022;22(1):125–136. doi:10.1016/j.acap.2021.04.016 33901729 PMC8542059

[79] Hansjürgens R. Soziale Arbeit in der Suchthilfe. In: Dettmers S, Bischkopf J, Altenhöner T, editors. *Handbuch gesundheitsbezogene Soziale Arbeit. 2. aktualisierte Auflage*. Ernst Reinhardt Verlag; 2021:186–197.

[80] Elms N, Link K, Newman A, Brogly SB. Need for women-centered treatment for substance use disorders: results from focus group discussions. *Harm Reduct J*. 2018;15(1):40. doi:10.1186/s12954-018-0247-5 30081905 PMC6080513

[81] Velleman R, Templeton LJ. Impact of parents’ substance misuse on children: an update. *BJPsych Adv*. 2016;22(2):108–117. doi:10.1192/apt.bp.114.014449

[82] Buth S, Bernard C, Schlömer H, Tödte M, Kalke J. *Kurzbericht Problematischer Substanzkonsum Und Vaterschaft*; 2016. Available from: https://www.bundesgesundheitsministerium.de/fileadmin/Dateien/5_Publikationen/Drogen_und_Sucht/Berichte/Kurzbericht_Problematischer_Substanzkonsum_und_Vaterschaft.pdf. Accessed January 23, 2024.

[83] Roozen HG, Boulogne JJ, Van Tulder MW, Van Den Brink W, De Jong CAJ, Kerkhof AJFM. A systematic review of the effectiveness of the community reinforcement approach in alcohol, cocaine and opioid addiction. *Drug Alcohol Depend*. 2004;74(1):1–13. doi:10.1016/j.drugalcdep.2003.12.006 15072802

[84] Dollinger B, Schmidt-Semisch H. Professionalisierung in der Drogenhilfe. In: Dollinger B, Schmidt-Semisch H, editors. *Sozialwissenschaftliche Suchtforschung. 1. Aufl. VS*. Verlag für Sozialwissenschaften; 2007:323–338.

[85] Helas I. Über den Prozess der Professionalisierung in der Suchtkrankenhilfe. In: Hausschild E, editor. *Suchtkrankenhilfe in Deutschland. Geschichte, Struktur Und Perspektiven*. Lambertus; 1997:147–161.

[86] Arnold T. *Zwischen Fachlichkeit und Fremdbestimmung: Eine rekonstruktive Annäherung an Soziale Arbeit in Suchtberatungsstellen*. Tectum Verlag; 2020.

